# Impact of Endodontic Irrigant Activation on Smear Layer Removal and Surface Disintegration of Root Canal Dentine In Vitro

**DOI:** 10.3390/healthcare11030376

**Published:** 2023-01-29

**Authors:** Matthias Widbiller, Andreas Rosendahl, Ralf Schlichting, Christine Schuller, Benedikt Lingl, Karl-Anton Hiller, Wolfgang Buchalla, Kerstin M. Galler

**Affiliations:** 1 Department of Conservative Dentistry and Periodontology, University Hospital Regensburg, 93053 Regensburg, Germany; 2 Independent Researcher, 94032 Passau, Germany; 3 Department of Operative Dentistry and Periodontology, Friedrich-Alexander-University Erlangen-Nuernberg, 91054 Erlangen, Germany

**Keywords:** smear layer, dentin, disinfection, ultrasonics, lasers, sodium hypochlorite, ethylenediaminetetraacetic acid

## Abstract

The objective of this study was to compare the ability of different endodontic irrigation activation methods to enable irrigant penetration, remove the smear layer from root canal walls after preparation, and investigate surface effects on dentine. Root canals of 90 single-rooted teeth were prepared and irrigated with EDTA (17%) and sodium hypochlorite (5%), where both irrigants or sodium hypochlorite only were activated as follows: conventional needle irrigation, ultrasonic activation, sonic activation (EDDY), or laser-based activation (photon-induced photoacoustic streaming/PIPS). For the evaluation of irrigant penetration into dentinal tubules, methylene blue was injected and activated as well. Subsequently, teeth were sectioned horizontally, and dye penetration depths were measured. Alternating sections were split in halves and randomly selected for scanning electron microscopic analysis. Root canal dentine was assessed for smear layer removal and surface disintegration according to a defined scoring system. The data were analyzed statistically with nonparametric and chi-squared tests for whole teeth and separately for coronal, middle, and apical thirds. All the tested activation methods removed a thicker smear layer than needle irrigation only. Additional activation of EDTA improved penetration depths of the irrigants, but not the smear layer removal. Surface disintegration of root canal dentine was observed with the additional activation of EDTA and particularly after laser-based techniques. Additional activation of EDTA does not seem to offer any convincing advantages in terms of irrigant penetration or smear layer removal but disrupts the dentine surface. Especially laser-based activation resulted in undesirable destruction of root canal wall dentine.

## 1. Introduction

In infected root canals, efficient biofilm removal and disinfection of the endodontic system can be considered a prerequisite for the resolution of periapical inflammatory processes and thus for the success of treatment [1]. The golden standard stipulates a combination of mechanical and chemical preparation, accompanied by activation of endodontic irrigation solutions, which can demonstrably increase their efficacy [2]. Different activation methods are available, among them ultrasonic, sonic, and laser-based techniques. The goal of activation is the induction of fluid movement and thus formation of shear stresses on root canal walls which can disrupt biofilms and improve smear layer removal [3]. The method that has been investigated most is ultrasonic activation, which appears to reliably support antimicrobial effects of the irrigation solution as well as the removal of smear layer and debris [2,3] and potentially aid in the healing of periapical lesions [4]. Sonic devices are easy to use and cost-effective and may also exert similarly beneficial effects [5]. Recently developed laser-based activation techniques by use of the middle infrared radiation spectrum have been advocated as promising tools for enhanced cleaning of the root canal system [6,7,8,9,10]. Two effects of fluid dynamics are responsible for the occurrence of enhanced fluid movement, namely acoustic streaming and cavitation. Acoustic streaming is induced by high-frequency oscillation of an object within the liquid, which will cause the movement. Cavitation, on the other hand, occurs if moving objects within the liquid create areas of high and low pressure, where the latter decreases the boiling point and thus allows for the formation of bubbles. These expand and later implode, causing rapid backflow and therefore movement of the liquid. Inside the root canal, the movement of the solution is hampered by the geometrical conditions of a long and narrow tube and further complicated by a potentially complex anatomy [11,12,13].

The clinical situation is quite intricate and involves a multitude of parameters such as root canal anatomy, length and diameter of the canal, microbiota and properties of the biofilm, as well as smear layer formation after mechanical preparation. Additional factors influence the efficacy of irrigation, among them the choice of the irrigation solution, concentration, flow rate, duration, and volume, as well as the properties of the applied instruments. A recent meta-analysis on ultrasonic activation noted that although there are numerous publications on this topic, it is difficult to make comparisons and draw accurate conclusions about the expected effects due to the large variety in terms of methodology [2]. The current study aimed at comparing different activation methods in terms of their ability to assist irrigant penetration and smear layer removal and, at the same time, assess changes or deterioration of the dentine surface. The null hypothesis stated that (i) no differences are expected in terms of the penetration depth of the irrigant and smear layer removal between different methods of activation and (ii) no surface effects will be observed on dentine after different treatment protocols.

## 2. Materials and Methods

### 2.1. Preparation of Teeth

The Ethics Committee of the University of Regensburg approved the use of extracted teeth (reference No. 19-1327-101; 20 February 2019). Informed consent was obtained from all the participants or, if the participants were under 16, from a parent and/or legal guardian. The study was planned and performed in accordance with the PRILE 2021 guidelines for laboratory studies in endodontology [14] and the Declaration of Helsinki.

Ninety single-rooted caries-free extracted human teeth with simple root canal anatomy (round or oval geometry, straight canal) were selected from a pool of extracted teeth and stored in 0.5% chloramine T-hydrate (Carl Roth, Karlsruhe, Germany). Dental radiographs were taken (vestibulo-oral and mesiodistal) to exclude any obstructions and verify the straight course of the root canal (curvature of 5° or less). The teeth were transferred into distilled water 24 h prior to experimentation. Preparation of standardized access cavities with a sufficiently large reservoir of 6 mm height, determination of working length, and root canal instrumentation were performed as described previously [8]. The root apex of each specimen was covered with a heavy condensation silicone impression material (Panasil tray Soft Heavy, Kettenbach, Eschenburg, Germany) to avoid extrusion of the irrigation solutions [15]. Root canals were instrumented with rotary files (Protaper Next Files X1 to X4 and X-Smart Plus Motor, Dentsply, Ballaigues, Switzerland) to size 40, 0.06 under irrigation with 5% sodium hypochlorite (NaOCl, SPEIKO, Münster, Germany), preheated to 60 °C, with a volume of 1 mL between the consecutive files. NaOCl was removed with paper tips, and the teeth were stored in distilled water before the final irrigation.

### 2.2. Final Irrigation and Activation

The irrigation solutions for the final irrigation were NaOCl (5%, 60 °C), distilled water (room temperature), and EDTA (17%, room temperature). For the final irrigation, the teeth were randomly assigned to conventional needle irrigation, ultrasonic, sonic, or laser-based activation (*n* = 15). The protocol for the final irrigation was designed as follows:

1. NaOCl (5 mL, 1 min);
2. Distilled water (5 mL, 1 min);
3. EDTA (5 mL, 1 min), with or without activation for 30 s (according to groups);
4. Distilled water (5 mL, 1 min);
5. NaOCl (5 mL, 1 min), with or without activation for 30 s, resting phase for 30 s, activation for 30 s (according to groups);
6. Distilled water (5 mL, 1 min);
7. Methylene blue, activation for 30 s.

In the conventional needle irrigation group (CNI), 1 mL of 5% NaOCl was injected without activation but a slight up-and-down movement of the cannula. During activated irrigation, EDTA was optionally activated and NaOCl was always activated. For ultrasonic irrigation (PUI), an NSK U File 25 (Irri S, NSK, Tochigi, Japan) was operated on an ultrasonic device (VDW, Munich, Germany) at 25% intensity. For sonic activation (EDDY), polyamide tips (EDDY, VDW, Munich, Germany) were inserted 1 mm short of working length and operated in combination with an air scaler (Sonicflex 2003/L, KaVo, Biberach, Germany) at 3.5 bar and level 3. Laser-based activation was performed in the PIPS (photon-induced photoacoustic streaming) mode on an Er:YAG laser (Lightwalker ATS, FOTONA, Ljubljana, Slovenia) at 20 mJ, 15 Hz, and 0.30 W.

**Figure 1:**
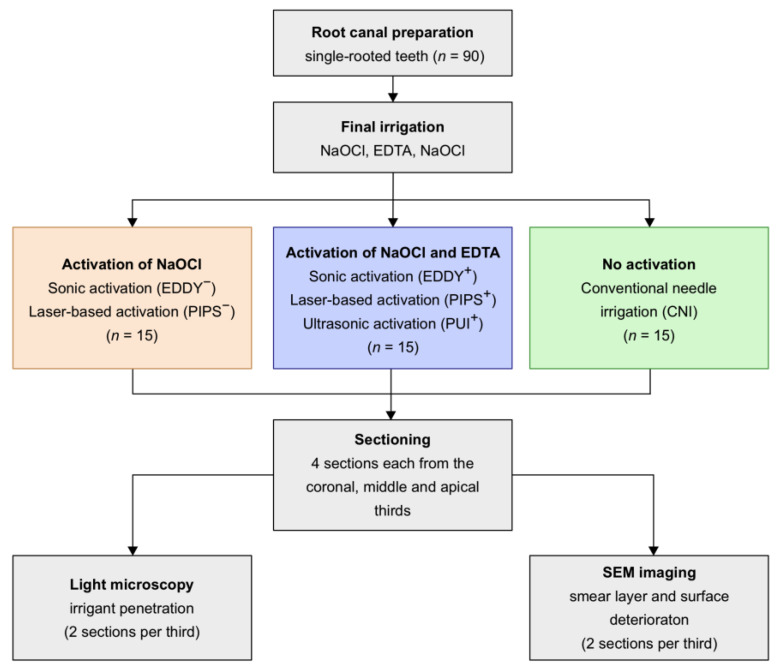
Flowchart of the experimental design and procedure.

For the evaluation of irrigant penetration, methylene blue was injected into the canals as the final step and activated in accordance with the protocol for the respective group. After the final irrigation, the root canals were dried with paper points and stored dry until further use. The information on the work flow, test and control groups, and the procedures for activation is summarized in Figure 1.

### 2.3. Sectioning and Imaging

Cross-sections of the treated teeth were prepared on a circular saw (Leitz 1600, Ernst Leitz Wetzlar, Wetzlar, Germany) under constant water cooling at 600 rpm with a feed speed of 0.01 m/min at a thickness of 300 µm. The sections were numbered consecutively and collected selectively from coronal, middle, and apical thirds of the root. Two non-consecutive sections per third were chosen and imaged under a light microscope with a corresponding camera system and software (Zeiss AXIO LAB A1 and ZEN core v2.0.66.1000, Jena, Germany). The images were introduced into Fiji [16] and the penetration depths of methylene blue into dentinal tubules were measured along 24 lines on a virtual clockface by two examiners as described before [8].

For scanning electron microscopic (SEM) imaging, the two remaining sections per third were broken into halves, the specimens were mounted onto aluminum stubs using self-adhesive carbon disks (Leit-Tabs, PROVAC, Sprendlingen, Germany) and sputter-coated with platinum. Two areas were randomly selected on each specimen and imaged using an FEI Quanta 400 environmental scanning electron microscope with a field emitter (FEI Europe B.V., Eindhoven, The Netherlands) operated in the low-vacuum scanning electron microscopy (LVSEM) imaging mode at a magnification of 800×.

Subsequently, the images were analyzed based on a scoring system for smear layer removal and surface deterioration. Untreated areas were excluded from further inspection so that the resulting scores strictly related to the surfaces after mechanical and/or chemical preparation. The following scoring system was used to assess both the smear layer removal and dentine surface deterioration:

- Score 0: not present;
- Score 1: detectable on ≤ 25% of the surface area;
- Score 2: detectable on 25–50% of the surface area;
- Score 3: detectable on 50–75% of the surface area;
- Score 4: detectable on > 75% of the surface area.

**Figure 2:**
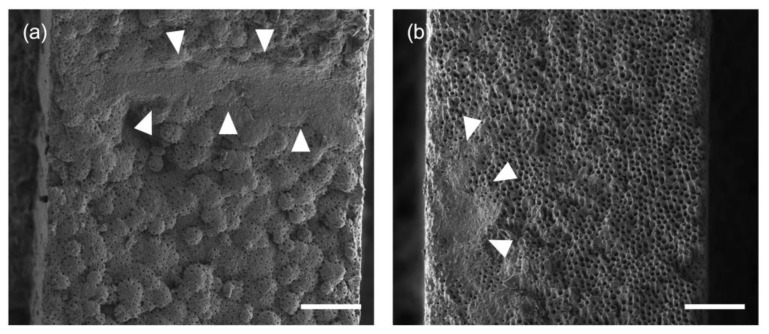
Root canal surfaces after treatment. (**a**) Native root dentine with the typical surface morphology and, in places, smear layer formation (arrows). (**b**) Deterioration and irregularities on the dentine surface after treatment and smear layer (arrows). Scale bar: 50 µm.

A high score reflects a larger coverage of the dentine by the smear layer after preparation and a high degree of surface deterioration, respectively. Furthermore, it is inherent to this scoring system that score 4 in terms of the smear layer, where most of the surface is covered, pairs with score 0 in terms of surface deterioration. An illustration of root canal surface structures important for scoring can be found in Figure 2.

### 2.4. Data Analysis

The maximum penetration depth per section was determined and medians with 25–75 percentiles were calculated for these values, both for whole root canals (all sections) as well as separately for coronal, middle, and apical segments. In case of smear layer removal, the median, and for surface deterioration, the maximum score from two images/section and two sections from coronal, middle, and apical segments were included into a frequency analysis. For whole teeth, the median score from all sections per tooth was included for smear layer removal, and the maximum score—for surface deterioration. The data were analyzed statistically with nonparametric tests (Mann–Whitney U) on an α = 0.05 level of significance to compare the groups in terms of penetration depths and score frequencies (SPSS, version 23.0, SPSS Inc., Chicago, IL, USA); *p*-values were adjusted familywise for multiple comparisons by the error rates method.

## 3. Results

### 3.1. Penetration Depth of an Irrigant with and without Activation of EDTA

**Figure 3:**
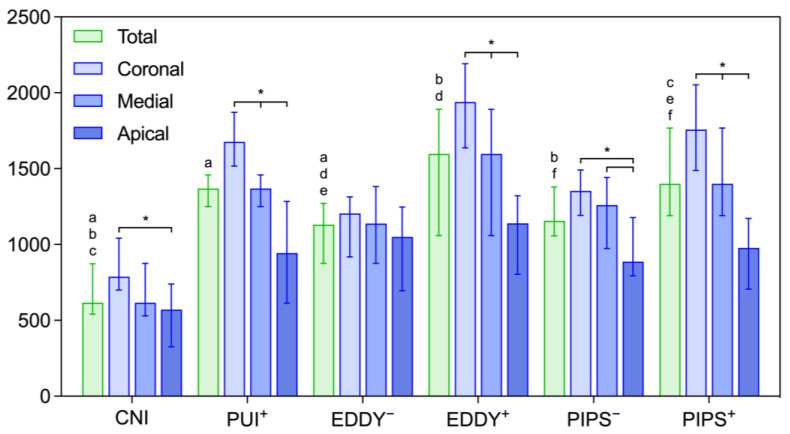
Penetration depths into dentinal tubules for the whole roots (total) and for the respective segments. Depicted are the medians and 25–75 percentiles (*n* = 15). CNI = conventional needle irrigation, EDDY = sonic activation, PUI = ultrasonic activation, PIPS = laser-based activation in the PIPS mode. A plus and minus symbol indicates whether EDTA has been additionally activated in the respective group or not. For the whole roots, the identical lowercase letters above the bars indicate statistically significant differences among them. The apical, medial, and coronal segments were compared with each other, and statistically significant differences were marked by asterisks.

Penetration depths for whole roots and for separate segments are depicted in Figure 3. The total penetration depths of an irrigant for conventional needle irrigation (616 µm) were significantly lower compared to all other groups (*p* = 0.000) but similarly high for all the activation methods (between 1131 µm and 1597 µm). The penetration of an irrigant was higher in the groups where EDTA was also activated; this difference was significant for both EDDY (*p* = 0.000) (1597 µm vs. 1131 µm) and laser-based PIPS (*p* ≤ 0.039) (1400 µm vs. 1156 µm). A separate consideration of penetration depths in the different root segments showed a decrease towards the apical third of the root. Overall, the penetration depths were highest for EDDY with activation of EDTA (coronal: 1940 µm; medial: 1598 µm; apical: 1140 µm) but low for EDDY without additional activation of EDTA (coronal: 1205 µm; medial 1139 µmv apical: 1050 µm). The reduction in penetration depth was significant from coronal to apical segments in all the groups except EDDY without EDTA activation (*p* ≤ 0.001).

### 3.2. Removal of the Smear Layer

**Figure 4:**
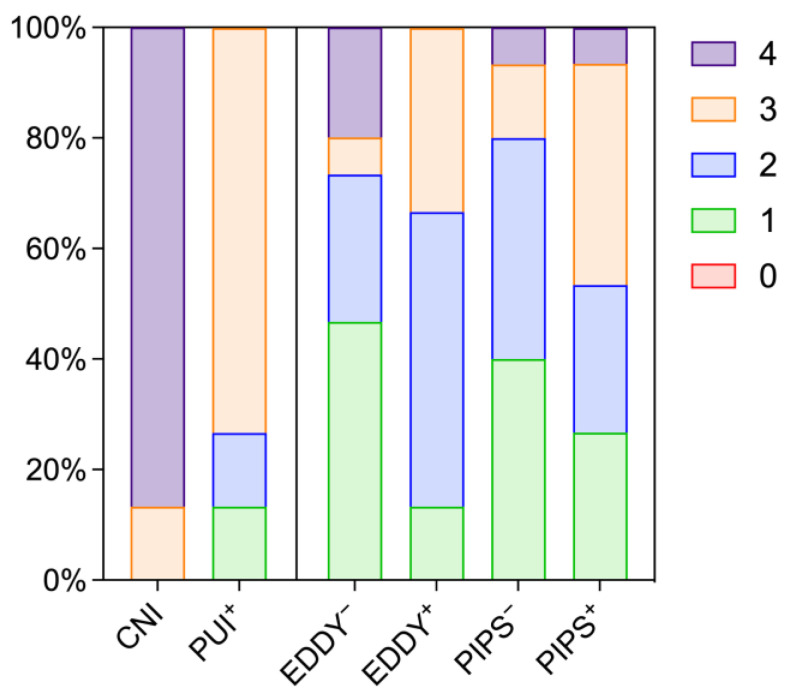
Scores for smear layer removal in whole tooth roots according to frequencies: 0 = not present, 1 = detectable on ≤ 25%, 2 = detectable on 25–50%, 3 = detectable on 50–75%, 4 = detectable on > 75% of the surface area. CNI = conventional needle irrigation, EDDY = sonic activation, PUI = ultrasonic activation, PIPS = laser-based activation in the PIPS mode. A plus and minus symbol indicates whether EDTA has been additionally activated in the respective group or not.

The effects of activation on smear layer removal are summarized in Figure 4 and detailed in Table 1 for whole roots as well as single segments. After conventional needle irrigation, 86.7% of the specimens presented with score 4, which indicates insufficient removal of the smear layer. In general, activation led to increased removal of the smear layer independent of the mode of activation, and an increasing amount of the remaining smear layer was observed from coronal towards apical segments. However, for EDDY as well as PIPS, additional activation of EDTA did not result in significantly improved smear layer removal (*p* > 0.250).

### 3.3. Deterioration of Dentine Surfaces

**Figure 5:**
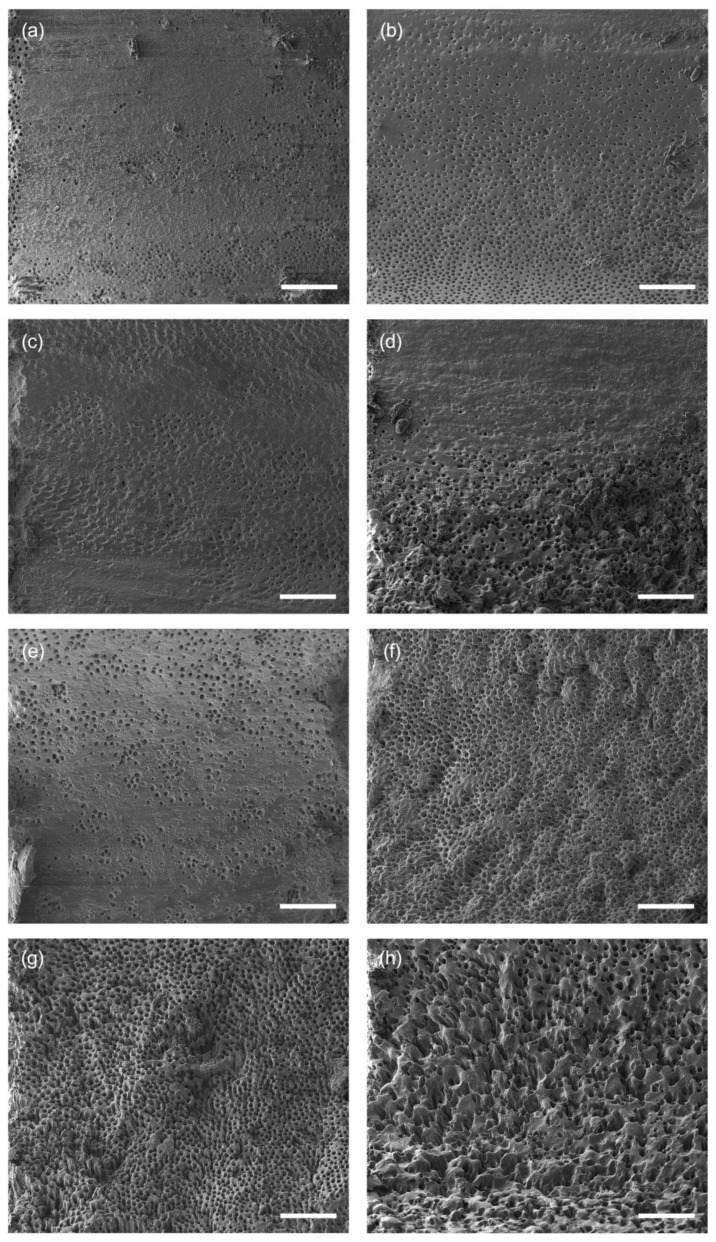
Representative images of dentine surfaces after treatment. (**a**) Conventional needle irrigation, (**b**) PUI^+^, (**c**) EDDY^−^, (**d**) EDDY^+^, (**e**) PIPS^−^, (**f**–**h**) PIPS^+^. Scale bar: 50 µm.

**Figure 6:**
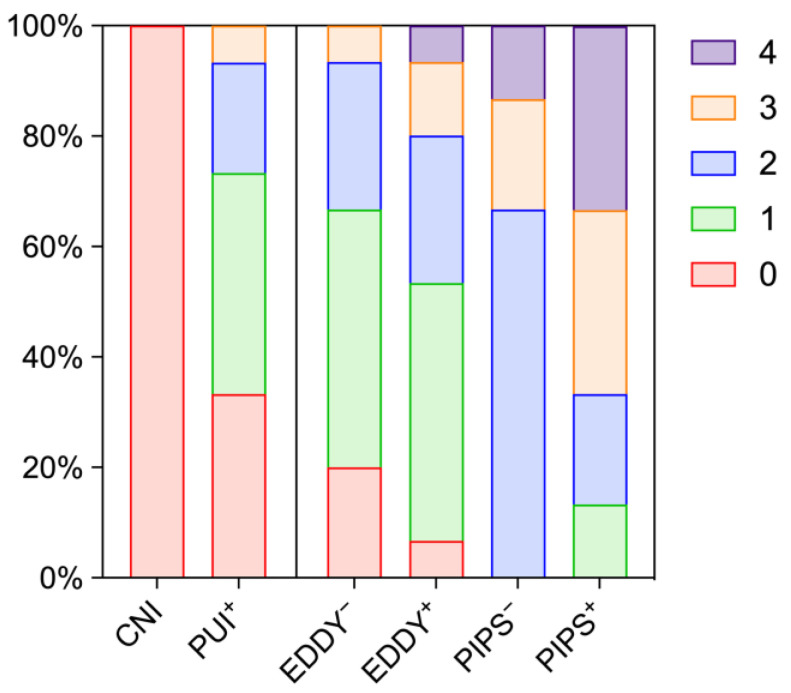
Scores for dentine surface deterioration in whole tooth roots according to frequencies: 0 = not present, 1 = detectable on ≤25%, 2 = detectable on 25–50%, 3 = detectable on 50–75%, 4 = detectable on >75% of the surface area. CNI = conventional needle irrigation, EDDY = sonic activation, PUI = ultrasonic activation, PIPS = laser-based activation in the PIPS mode. A plus and minus symbol indicates whether EDTA has been additionally activated in the respective group or not.

The effects of activation on dentine surfaces are illustrated by representative images in Figure 5 and summarized in Figure 6 and Table 2. Root canal walls from the specimens after conventional needle irrigation showed significantly less deterioration as observed by surface irregularities compared to the activation groups (*p* ≤ 0.001). Compared to PIPS, the use of PUI and EDDY appeared less damaging, where PUI with additional activation of EDTA resulted in similar scores compared to EDDY without it. The additional activation of EDTA increased the degree of surface deterioration both for EDDY and PIPS, however not significantly (*p* > 0.217). In contrast to smear layer removal, there was no clear tendency from the coronal to the apical segments in surface destruction.

## 4. Discussion

In this study, three important parameters that influence chemo-mechanical preparation of the root canal were evaluated depending on the mode of irrigant activation, namely the penetration depth of the irrigant into dentinal tubules, the removal of the smear layer, and the integrity of the dentine surface. The tested modes of activation included conventional needle irrigation as a control, and ultrasonic, sonic, and laser-based activation in test groups. These methods are commonly used during endodontic treatment, in particular, sonic and ultrasonic activation, based on a sound level of evidence [2]. Laser-based PIPS has been of much interest lately, as the effects on cleaning and disinfection appear promising [6,7,8,9]. A systematic comparison of laser-based activation with sonic and ultrasonic activation showed that the use of this method may be beneficial; however, heterogenous study designs make a clear statement complicated [17]. Systematic reviews have shown that ultrasonic activation leads a to significantly improved removal of the smear layer and debris compared to needle irrigation [2], that different methods of activation result in reduced postoperative pain and improved cleanliness of the canal [18], and that ultrasonic activation improves bacterial reduction [19]. Sonic activation appears to achieve similar results with regard to the removal of the smear layer and debris compared to ultrasonic activation [5]. Laser-based PIPS seemed effective in removing the smear layer and improving irrigant penetration [6,8]; furthermore, it offers easy handling, as the glass fiber tip is confined to the coronal chamber above the orifice, and insertion into the canal itself is not required.

With the current study, the parameters of irrigant penetration into dentinal tubules, removal of the smear layer, and the state of the dentine surface after treatment were evaluated. Whereas the former two have been investigated and discussed in several publications, surface effects have not been assessed in detail and, in particular, not in a systematic way, where a total amount of 24 areas per root canal were chosen for SEM analysis. The initial null hypothesis of no differences in irrigant penetration, smear layer removal and no surface effects must be rejected on the basis of the results presented.

Penetration of irrigant solutions into dentinal tubules is desirable and may affect the outcome after endodontic treatment. Histologic studies have shown that the median penetration depths of bacteria into dentinal tubules amount to 390 µm [20]. Thus, the penetration depths that were achieved in this study may be clinically relevant. Enhanced irrigant penetration after activation is to be expected and confirms data from previous studies [21,22]. The use of single-rooted teeth with a rather simple root canal geometry and an extensive irrigation protocol may have contributed to the large penetration depths observed in this study. While there is consensus that the use of sodium hypochlorite throughout instrumentation and the final irrigation with EDTA for the removal of the smear layer are beneficial [23], specific and evidence-based recommendations in respect of irrigation and activation during endodontic treatment are currently lacking. A rinse with sodium hypochlorite after smear layer removal with EDTA may enhance the penetration into dentinal tubules and thus the elimination of bacteria [24], but it will also enhance dentine erosion [25]. Controversies can be found with regard to the activation of EDTA. Most studies follow a protocol where only sodium hypochlorite is activated. However, early investigations on laser-based activation included the activation of EDTA in their study protocols [6], which led to increased penetration depths of the final irrigant into dentinal tubules. Increased levels of energy and, thus, streaming of the solution may explain this effect. Interestingly, the penetration depths that were achieved in this study were highest for EDDY, which is the mode of activation which inserts the least amount of energy among the tested methods, however, only in the group where additional activation of EDTA was carried out.

While sodium hypochlorite is still the gold standard for tissue dissolution and disinfection during root canal treatment, chemo-mechanical preparation results in a smear layer that contains collagen, hydroxyapatite, and bacteria, which covers the instrumented surface of the root canal. EDTA is commonly used to remove the smear layer due to its properties to bind calcium and thus remove the inorganic phase and therefore lead to ablation of the smear layer from the dentine surface [24]. With conventional needle irrigation, the use of EDTA appears to have rather little effect, where score 4 (more than 75% of the surface remains covered with the smear layer) was present in most of the specimens. In general, irrigant activation enhanced smear layer removal independent of the mode of activation. Yet, considerable amounts of the smear layer remain on the dentine surface, which may affect subsequent disinfection and obturation. While clean dentine surfaces with visible dentinal tubules can be seen in many SEM studies after the use of EDTA, it has to be emphasized that the strengths of this study lie in the random and numerous selections of areas within the root canal chosen for analysis, which enables a much more objective evaluation of the effectiveness of the tested methods.

Whereas both EDDY and PIPS were tested with and without activation of EDTA, ultrasonic activation was carried out only with activation of EDTA. This was based on the hypothesis that PUI with concomitant EDTA activation was an invasive approach due to the amount of energy transmission and potential direct contact of the metal instrument with the root canal wall and, thus, could serve as a positive control. Surprisingly, EDDY as a comparatively soft polyamide device and, in particular, PIPS activation as a no-touch approach revealed larger areas of surface destruction. PUI with activation of EDTA even resulted in similar surface effects as EDDY without activation of EDTA, and PIPS without EDTA activation had more deleterious effects on surface integrity than PUI with the respective activation step. It may be deduced that PUI is the least destructive activation method in this respect, whereas laser-based activation leads to considerable surface disintegration. However, applications of EDDY and PIPS were very efficient in removing the smear layer even without activation of EDTA. In view of the observation that additional activation only moderately improved the penetration of the irrigants and smear layer removal within the individual activation methods, the benefit of EDTA activation must be challenged against the background of possible dentine deterioration.

The effect of PIPS on dentine surface integrity had not been investigated systematically before. DiVito et al. described “minimally disruptive effects on the canal walls”, did not observe thermal damage, and rated the accompanying changes as noncritical [6]. Even though this was not observed in this study, it cannot be excluded that thermal effects may still occur locally. More explicit than the scores are SEM images, which show an uneven, rough surface, fractured dentine chips. While uncontrolled removal of dentine has to be considered as critical and undesirable, questions also arise concerning the final obturation. Fragile peaks and elevations that fracture under the mechanical stress applied during root canal filling may result in an unfavorable interface between the dentine, the sealer, and the gutta-percha. Therefore, more attention should be paid to the effects of different activation methods on root wall dentine and to the quality of subsequent obturation.

## 5. Conclusions

Irrigant penetration depths were similarly high for ultrasonic, sonic, and laser-based activation and significantly higher than for conventional needle irrigation. The activation of EDTA increased penetration depths, but also caused undesirable surface effects. Laser-based PIPS led to pronounced deterioration of the dentine surface, which should be considered as a drawback for this method of activation.

## Figures and Tables

**Table 1:** Smear layer removal median. Frequencies of scores in % (0 = not present, 1 = detectable on ≤25%, 2 = detectable on 25–50%, 3 = detectable on 50–75%, 4 = detectable on >75% of the surface area). In the statistical analysis, significant differences were observed between CNI and each of the test groups (*p* = 0.000). The differences between EDDY and PIPS with and without activation of EDTA were not significant (*p* > 0.250).

	Score	CNI	PUI^+^	EDDY^−^	EDDY^+^	PIPS^−^	PIPS^+^
Total	0	0.0	0.0	0.0	0.0	0.0	0.0
	1	0.0	13.3	46.7	13.3	40.0	26.7
	2	0.0	13.3	26.7	53.3	40.0	26.7
	3	13.3	73.3	6.7	33.3	13.3	40.0
	4	86.7	0.0	20.0	0.0	6.7	6.7
Coronal	0	6.7	0.0	0.0	0.0	0.0	6.7
	1	0.0	6.7	40.0	33.3	40.0	46.7
	2	26.7	46.7	33.3	40.0	33.3	20.0
	3	0.0	40.0	13.3	20.0	20.0	20.0
	4	66.7	6.7	13.3	6.7	6.7	6.7
Medial	0	0.0	0.0	0.0	0.0	6.7	6.7
	1	0.0	13.3	60.0	33.3	33.3	26.7
	2	13.3	26.7	20.0	40.0	40.0	20.0
	3	0.0	60.0	6.7	20.0	13.3	40.0
	4	86.7	0.0	13.3	6.7	6.7	6.7
Apical	0	0.0	0.0	0.0	0.0	13.3	0.0
	1	0.0	13.3	13.3	0.0	26.7	13.3
	2	0.0	6.7	26.7	20.0	33.3	13.3
	3	13.3	46.7	33.3	60.0	0.0	40.0
	4	86.7	33.3	26.7	20.0	26.7	33.3

**Table 2:** Surface deterioration maximum. Frequencies of scores in % (0 = not present, 1 = detectable on ≤25%, 2 = detectable on 25–50%, 3 = detectable on 50–75%, 4 = detectable on >75% of the surface area). In the statistical analysis, significant differences were observed between CNI and each of the test groups (*p* = 0.000). The differences between EDDY and PIPS with and without activation of EDTA were not significant (*p* > 0.217).

	Score	CNI	PUI^+^	EDDY^−^	EDDY^+^	PIPS^−^	PIPS^+^
Total	0	100.0	33.3	20.0	6.7	0.0	0.0
	1	0.0	40.0	46.7	46.7	0.0	13.3
	2	0.0	20.0	26.7	26.7	66.7	20.0
	3	0.0	6.7	6.7	13.3	20.0	33.3
	4	0.0	0.0	0.0	6.7	13.3	33.3
Coronal	0	100.0	46.7	46.7	20.0	13.3	6.7
	1	0.0	40.0	33.3	66.7	13.3	13.3
	2	0.0	13.3	20.0	6.7	53.3	33.3
	3	0.0	0.0	0.0	6.7	13.3	20.0
	4	0.0	0.0	0.0	0.0	6.7	26.7
Medial	0	100.0	40.0	26.7	13.3	20.0	13.3
	1	0.0	46.7	53.3	53.3	46.7	26.7
	2	0.0	13.3	13.3	13.3	26.7	6.7
	3	0.0	0.0	6.7	20.0	6.7	33.3
	4	0.0	0.0	0.0	0.0	0.0	20.0
Apical	0	100.0	46.7	53.3	33.3	13.3	13.3
	1	0.0	33.3	33.3	46.7	40.0	33.3
	2	0.0	13.3	6.7	13.3	26.7	20.0
	3	0.0	6.7	6.7	0.0	13.3	26.7
	4	0.0	0.0	0.0	6.7	6.7	6.7

## Data Availability

The data presented in this study are available on request from the corresponding author.
